# SLAMF7: A Potential Target for CAR T-Cell Therapy in Multiple Myeloma

**DOI:** 10.3390/cancers17213471

**Published:** 2025-10-29

**Authors:** Caterina Alati, Martina Pitea, Gaetana Porto, Erica Bilardi, Maria Bruna Greve, Iolanda Donatella Vincelli, Andrea Rizzuto, Giorgia Policastro, Maria Eugenia Alvaro, Giovanna Utano, Demetrio Gerace, Alessandro Allegra, Eugenio Piro, Marco Rossi, Massimo Martino

**Affiliations:** 1 Hematology and Stem Cell Transplantation and Cellular Therapies Unit (CTMO), Department of Hemato-Oncology and Radiotherapy, Grande Ospedale Metropolitano “Bianchi-Melacrino-Morelli”, 89133 Reggio Calabria, Italy; 2 Stem Cell Transplant Program CIC587, 89133 Reggio Calabria, Italy; 3 Hematology Unit, Department of Human Pathology in Adulthood and Childhood “Gaetano Barresi”, University of Messina, 98125 Messina, Italy; 4 Department of Hematology-Oncology, Dulbecco University Hospital, 88100 Catanzaro, Italy

**Keywords:** multiple myeloma, CAR-T cell therapy, SLAMF7, new CAR-T targets, BCMA, elotuzumab, dual CAR-T

## Abstract

Signaling Lymphocyte Activation Molecule Family member 7 (SLAMF7) represents a potential target for CAR T-cell therapy in the treatment of multiple myeloma (MM), and it is a promising alternative to classic BCMA-CAR therapy. The receptor is expressed on immune cells, particularly natural killer cells and T cells, that can trigger both activating and inhibitory signals. It is highly expressed in MM cells at all disease stages, playing a crucial role in cell adhesion and communication between immune cells, and being involved in the development and progression of the disease. The target has a proven clinical success with Elotuzumab (anti-SLAMF7 antibody); it works by activating immune cells to kill myeloma cells, and limited expression on normal tissues, with, potentially, few side effects. SLAMF7’s combination of specificity, stability, and clinical validation makes it an excellent target for current and future MM therapies. However, ‘fratricide death’, a phenomenon where the engineered CAR-T cells attack and kill each other, is a critical issue that requires safe engineering solutions. In this work, we will provide an overview on the field with a specific focus on SLAMF7 as an emerging CAR-T cell target in MM.

## Simple Summary

SLAMF7 is highly expressed on myeloma cells at all disease stages, with a limited expression on normal tissues, and, potentially, with few side effects. It works by activating immune cells to kill myeloma cells and could represents a platform for next-generation therapies, such as CAR-T cells, bispecific antibodies, and antibody–drug conjugates. SLAMF7’s combination of specificity, stability, and clinical validation makes it an excellent target for current and future multiple myeloma therapies.

## 1. Introduction

Multiple myeloma (MM) constitutes 1–2% of all cancers and 10–15% of haematological malignancies [1], with an estimated incidence of 4.5–6.0 cases per 100,000 persons per year, in individuals with a median age of 65–70 years [2]. MM has experienced a significant enhancement in survival outcomes over the past two decades, which is indicative of the advancements in medical research and treatment options [3]. Cellular immunotherapies, in particular anti-BCMA autologous chimeric antigen receptor (CAR) T-cell therapy, have played a pivotal role in this progress [4]. The anti-BCMA CAR-T idecabtagenevicleucel (Ide-cel) and ciltacabtageneautoleucel (Cilta-cel), represent a significant development in the field of cancer therapy, as they are the first to be approved for use in patients with relapsed (R) or refractory (R) MM [5,6,7,8]. Despite the initial promise of successful outcomes, most patients ultimately experience disease relapse or progression, with a 5-year overall survival (OS) rate of approximately 60%. Only 10–15% of patients achieve the expected survival rate of the general population [9,10]. Consequently, in the current state of knowledge, MM can still be regarded as an incurable disease. CAR-T cell therapy for MM has several limitations. One is the risk of relapse due to antigen loss or tumour heterogeneity, whereby cancer cells no longer express the target antigen or exhibit significant variation; patients may also experience severe side effects; and the manufacturing process is complex and costly, and further research is needed to improve durability and broaden applicability.

## 2. SLAMF7 Target

Signaling Lymphocytic Activation Molecule Family Member 7 (SLAMF7) is a cell surface protein expressed on natural killer (NK) cells and in over 95% of malignant plasma cells (mPCs) [11,12]. Its role in PC survival renders it an attractive target for therapeutic intervention. The SLAM family of receptors, which are expressed on hematopoietic cells, plays a crucial role in regulating the immune system [13,14]. The SLAM family comprises six members: CD229, SLAM, NTB-A, 2B4, CD84, and CS1. SLAMF7, also known as CS1/CRACC/CD319, has been shown to promote the proliferation and growth of MM cells due to its high expression in these cells [15]. Furthermore, the expression of this protein is also observed in B cells, T cells, dendritic cells, NK cells, and monocytes. SLAMF7 has been implicated in various biological processes, including cell viability, humoral immunity, autoimmunity, cell adhesion, and lymphocyte development. SLAMF7 plays a crucial role in the pathogenesis of MM, and it possesses unique characteristics that make it a promising target for immunotherapeutic interventions [16]. Novel therapeutic interventions that target SLAMF7 include elotuzumab (ELO), a humanized IgG1 kappa monoclonal antibody [17], and CAR-T [18,19]. ELO, a humanized IgG1 kappa monoclonal antibody, activates NK cells directly and through antibody-dependent cellular cytotoxicity [12,17]. ELO enhances SLAMF7-SLAMF7 interactions between NK cells and myeloma cells, sending costimulatory signals that enhance killing. While ELO has minimal clinical efficacy as a single agent, it is effective in combination with lenalidomide and dexamethasone [20]. CAR T cells require lower antigen density for activation than antibodies do [21]. This is a critical difference: antigen downregulation below the CAR activation threshold leaves the T cell silent, rendering CAR T cell therapy ineffective. The binding affinity of the antigen recognition domain substantially affects the sensitivity of CAR T cells to antigens. The threshold for CAR T cell activation is inversely correlated with scFv affinity [22]. Even though SLAMF7 CAR T cells use the same targeting domain as ELO, the CAR format allows recognition at lower antigen densities. When CAR T cells meet their targeted antigen on a cell’s surface, the cells bind to it, become activated, and proceed to proliferate and become cytotoxic. They kill tumor cells directly through multiple mechanisms, including cytotoxicity and cytokine secretion. Each CAR T cell can also proliferate extensively after encountering antigen, creating a self-amplifying effect. These mechanistic differences mean that CAR T cells are potentially more potent, but they also carry risks of more severe side effects, such as cytokine release syndrome (CRS). CAR T-cells can eliminate tumor cells that might resist antibody therapy due to their lower antigen density threshold and direct cytotoxicity. However, these same properties increase the risk of on-target/off-tumor toxicity against normal SLAMF7+ cells.

The present article will evaluate the potential of SLAMF7 as a CAR-T target in MM, emphasizing the potential role in the advancement of treatment options and instilling a sense of hope and optimism in the audience.

## 3. The Challenges and Limitations of CAR-T Therapy in Multiple Myeloma

CAR-T in MM is a revolutionary therapy, albeit not yet definitive. Indeed, while offering a valuable therapeutic option for heavily pretreated patients, its durability is still evolving, thus not curative for most patients. The treatment has been shown to have a high initial response rate (RR) (e.g., >70% ORR in Ide-cel and Cilta-cel trials), although many patients relapse within 12–18 months [4,5,6,7,8]. However, the ongoing research and development in the field of CAR-T therapy provide reassurance about the continuous efforts to improve MM treatment.

Commercial CAR-T therapies in MM target BCMA [23,24]. Despite the dramatic responses produced by CAR-T cell therapy, most patients will eventually progress. The reasons for this are multifactorial. The hypothesis that tumour cells may downregulate or even lose BCMA has been proposed as a mechanism leading to relapse due to immune evasion [25,26,27]. Several other factors must be considered, including the exhaustion of CAR-T cells, the presence of an immunosuppressive tumor microenvironment, and the persistence of myeloma stem-like cells [28]. CAR-T cell exhaustion, characterized by poor persistence and dysfunction due to persistent antigen stimulation and an immunosuppressive tumour environment, further complicates following treatments [29]. Prior antimyeloma therapy has been identified as a host-related factor influencing CAR-T response, resulting in impaired T cell function and a microenvironmental deficit [30]. Tumor-intrinsic resistance mechanisms have been shown to include complex genomic alterations and immunomodulatory gene mutations [31]. Systemic inflammation prior to CAR-T administration has been identified as a potent biomarker of response, reflecting a pro-inflammatory tumour microenvironment characterized by regulatory T-cell populations and myeloid-derived suppressor cells [32,33].

**Figure 1:**
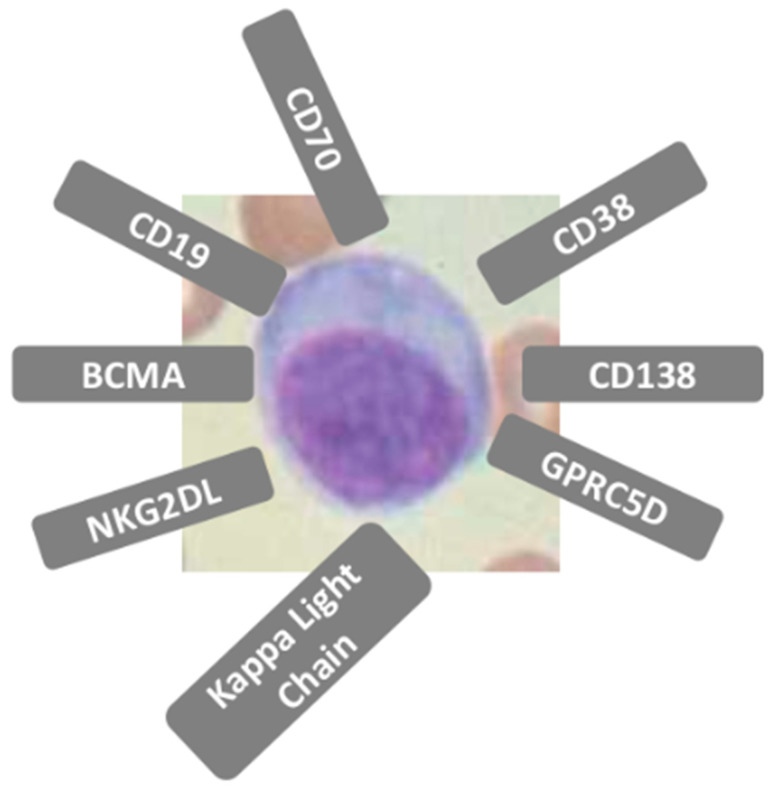
Multiple myeloma cells express a variety of surface antigens. Expression patterns of these and other antigens can be used for diagnosis, monitoring, and have prognostic value.

Putative targeting molecules identification other than BCMA is a key area of clinical study and development [34,35,36]. MM cells express a variety of surface antigens (Figure 1 and Table 1). Expression patterns of these and other antigens can be used for diagnosis, monitoring, and have prognostic value The main targets under study are summarized in Table 2, with their biological characteristics and the pros and cons of their use [37,38,39,40]. While most targets are still in the preclinical stage, only a small number are currently being explored in clinical trials. GPRC5D has been identified as a promising therapeutic target due to its high expression levels in myeloma cells, in contrast to its minimal expression in normal tissues, with an expression restricted primarily to hair follicles, hard keratinizing tissue (hair shaft, nail, tongue center), and a subset of cells in skin [41]. In a recently published meta-analysis, 130 MM patients treated with GPRC5D-targeted CAR T-cell therapy demonstrated an ORR of 87%, with 74% of these patients having received prior BCMA-targeted therapy. The proportion of patients achieving a partial response (PR) was 25%, while 33% achieved a very good partial response (VGPR), and 48% achieved a complete/stringent complete response (CR/sCR). Furthermore, 65% of patients achieved minimal residual disease (MRD) negativity [42]. These results suggest that GPRC5D is an attractive target in the treatment of R/R MM and heavily pretreated patients. GPRC5D is independent of BCMA expression patterns on myeloma cells, and it appears to be the ideal candidate in the relay of myeloma treatment at relapse after anti-BCMA therapies. SLAMF7 is a valid target but is less clinically advanced, with ongoing trials still evaluating its full potential. While individual studies show promising results for each, such as a high ORR for SLAMF7-CAR-T and high CR rates for both BCMA and GPRC5D-CAR-T, a direct head-to-head comparison to determine which is superior is currently not available. 

Another strategy, which is still under investigation, involves dual-targeting of CARs [43]. Many ongoing investigations are to evaluate dual-targeting strategies with various combinations based on BCMA, CD19, CD138, and SLAMF7, together or in sequence, and some already showed improved clinical activity in early phase clinical studies [44]. Regarding treatment approaches to augment anti-MM activity of a single targeted immunotherapeutic agent or to overcome an immunosuppressive BM microenvironment, a combination of different anti-MM agents with distinct mechanisms of action remains very attractive strategies. The primary objective of this approach is twofold: to enhance tumour coverage and to minimize antigen loss. 

CAR-T cell exhaustion prevention is explored through the optimization of CAR-T cell structure, the utilization of early memory T cells, and the inhibition of intracellular exhaustion-related signals through genetic modifications or inhibitors [45]. The enhancement of tumor activity of CAR-T cells and central memory CAR-T cells can be achieved through genetic modifications, such as the incorporation of immune-stimulatory receptors and the deletion of genes associated with CAR-T cell anergy. Research has demonstrated that armored CAR-T cells, which secrete cytokines or express pro-inflammatory ligands, have the capacity to reshape the tumor microenvironment [46]. Overcoming immunosuppressive cells in the bone marrow is crucial in the management of MM. These cells, including osteoclasts, myeloid-derived suppressor cells, tumor-associated macrophages, regulatory T cells, regulatory B cells, tumor-associated neutrophils, and bone marrow stromal cells, engage in signal integration with MM cells, facilitating their survival and proliferation. In addition, these cells have been shown to inhibit the activity of effector T cells, thereby enabling MM cells to evade immune surveillance.

Another significant issue is toxicity, and CRS and immune effector cell-associated neurotoxicity syndrome (ICANS) are prevalent [47]. The incidence of CRS ranges from 80% to 90% among patients, predominantly manifesting as mild symptoms, though in some instances, it can be severe; ICANS is less prevalent but can be life-threatening [48]. Prolonged cytopenias, infections, and hypogammaglobulinemia represent additional risks [49].

Manufacturing time and production failure represent additional factors that contribute to this phenomenon [50]. Autologous (auto) CAR-T production requires 3–5 weeks (“vein-to-vein” time), and patients with aggressive disease may experience disease progression during this period. The risk of manufacturing failure is high if the T cells collection is impaired due to excessive pretreatment [51]. Based on these considerations, CAR-T therapy is currently available only in specialized centers that require highly trained staff, intensive monitoring, and inpatient capacity for toxicity management. Access is especially limited in low- and middle-income countries [52].

## 4. SLAMF7-CAR-T Construct

A SLAMF7-CAR-T construct has been developed in Germany, and the preclinical results were published in 2017 [53]. The construct is composed of a lentiviral vector that contains a targeting domain derived from the monoclonal antibody targeting SLAM7, huLuc63, fused to an IgG4-Fc Hinge-CH2-CH3 spacer with a 4/2NQ modification to prevent binding of Fc-receptors, a CD28/CD3zeta signaling module in cis with a T2A element, and a truncated epidermal growth factor receptor (EGFRt). This construct was selected for its ability to reduce FcγR binding and prevent off-target activation. SLAMF7 CAR T-cells were successfully prepared using CD8+ and CD4+ T-cells from MM patients and healthy donors. In vitro, the efficacy of the drug was demonstrated using SLAMF7 myeloma cell lines, with 80% and 90% lysis observed after 4 h and 20 h, respectively [53]. Furthermore, the drug promoted productive proliferation, and no differences were observed between newly diagnosed and RR MM.

CAR-T cells have been shown to selectively eliminate SLAMF7+high lymphocytes, including NK cells, CD4+ and CD8+ T cells, and B cells, while preserving the function of other lymphocytes. The internalization of the SLAMF7 protein has been demonstrated to reduce expression, thereby enabling CD8+ and CD4+ cells to acquire a low SLAMF7 phenotype [14]. A CD8+ fratricide-resistant SLAMF7 CAR-T was developed, thereby ensuring the preservation of functional CD8+ T cell cultures. Fratricide death occurs because SLAMF7 is expressed on the CAR-T cells themselves, leading them to attack each other during manufacturing and after infusion. Given that SLAMF7 is expressed in NK cells, this therapy can result in cell death and deficiencies, potentially leading to severe infections. A suicide gene within the SLAMF7 CAR-T encodes a dimerization domain containing a caspase-9 domain, which is efficacious in the high-tumor-burden myeloma model, despite the fratricide of CD8+CS1-expressing CAR-T cells [13]. This approach facilitates the elimination of SLAMF7 CAR-T cells prior to NK cell depletion, thereby substantially enhancing the development of antitumor activity [53].

## 5. SLAMF7-CAR-T Clinical Trials

Several researchers have developed anti-SLAMF7 CAR T cells and tested them in preclinical models [14,22,53]. Gogishvili et al. developed an anti-SLAMF7 CAR T cell construct using the target-binding domain of ELO paired with a CD28 co-stimulatory domain [53]. O’Neal et al. [14] generated anti-SLAMF7 CAR T cells with a different target-binding epitope (the distal V2 domain) and a third generation CD28 and 4-1BB combination co-stimulatory domain. These cells effectively killed malignant plasma cells [22]. These CAR T cells were predominantly CD4+, suggesting that CD8+ CAR T cells were destroyed during ex vivo production. Consequently, they used CRISPR/Cas9 technology to delete SLAMF7 and create anti-SLAMF7 CAR T cells that were resistant to fratricide. Despite yielding a more balanced CD4:CD8 T cell profile, SLAMF7-deficient CAR T cells were not significantly more effective in murine xenograft models. Overall, while the consequences of fratricide for anti-SLAMF7 CAR T cells and other immune cells require further investigation, these studies suggest that anti-SLAMF7 CAR T cells could be a promising therapeutic strategy for treating myeloma.

### 5.1. Autologous Approaches

As previously indicated, SLAMF7 is expressed on NK cells, and a subset of CD8+ T cells. The depletion of these leukocyte subpopulations, induced by drugs targeting the antigen, has been demonstrated to result in an increased risk of infection [14]. Incorporate a suicide gene to enhance safety, particularly with respect to the risk of infectious complications. In the aforementioned “A Phase I Clinical Trial of T-cells Expressing an Anti-SLAMF7 CAR for Treating Multiple Myeloma,” the authors have engineered a novel anti-SLAMF7 CAR capable of specifically recognizing and eradicating SLAMF7-expressing tumors in murine models. The protocol involves the genetic modification of autologous T cells with genes that encode an inducible caspase 9 (IC9) cell suicide system, in conjunction with the anti-SLAMF7 CAR-T cell therapy [54]. The administration of the dimerizer drug Rimiducid is crucial for activating the IC9 suicide gene, thereby leading to the eradication of CAR T cells. In this protocol, the researchers employed the suicide gene system to eliminate CAR-T cells in the event of severe toxicities [55]. One potential drawback is the premature elimination of CAR-T cells [56]. If the dimerizer drug is administered too early or by accident, the therapeutic CAR-T cells may be eliminated before they can fight the tumor, which would compromise the treatment’s effectiveness. Once activated, the suicide mechanism cannot be reversed, necessitating additional CAR-T infusions if the tumor persists. Activation eliminates the potential for durable CAR-T cell persistence, which is crucial for preventing relapse.

CARAMBA-1 is a pioneering clinical trial of adoptive immunotherapy with auto SLAMF7 CAR-T cells in patients with advanced MM who have exhausted conventional therapies [57]. The CARAMBA-1 clinical trial is an open-label, non-randomized, multicenter study that combined a phase I dose-escalation with a phase II dose-expansion part to assess the feasibility, safety, and antimyeloma activity of SLAMF7 CAR-T cells [58]. The trial distinguishing characteristic is its provision of clinical evidence substantiating the notion of virus-free CAR gene transfer through the implementation of advanced “Sleeping Beauty” transposon technology [59]. The transposition of Sleeping Beauty in CAR-T engineering is a compelling prospect, given the high rate of stable CAR gene transfer facilitated by optimized hyperactive SB100X transposase and transposon combinations, encoded by mRNA and minicircle DNA, respectively, as preferred vector embodiments [60]. This approach has the potential to facilitate and expedite vector procurement, CAR-T manufacturing, and distribution, and promises to provide a safe, effective, and economically sustainable treatment. Its main advantages over viral systems like lentiviruses are its non-viral nature, potentially lower manufacturing cost and faster production timeline. CARAMBA cell manufacturing takes 14 days in total. Starting from an unmobilized leukapheresis targeting ≥5 × 10^9^ total white blood cells, the leukocytes are split so that both portions will contain approximately similar total numbers of CD4+ and CD8+ T cells. The CARAMBA is the first clinical trial with virus-free CAR-T cells in Europe, and the first clinical trial that uses advanced sleeping beauty technology worldwide.

### 5.2. Allogeneic (‘Off-the-Shelf’) Approaches

The first allo-CAR-T anti-MM to be tested within a clinical trial is UCARTCS1, an off-the-shelf SLAMF7-targeting allo-CAR T-cell product derived from healthy donors. The product was manufactured using TALEN gene editing technology to eliminate endogenous expression of T-cell receptor (TCR) and SLAMF7 [61,62]. The product has been shown to inhibit the TRAC gene, thereby preventing GVHD by disrupting TCR assembly. The product also knocks out SLAMF7 to facilitate robust expansion and yield, while avoiding the “fratricide” effect. UCARTCS1A is equipped with an RQR8 safety switch, which functions as a CD20 mimotope. If necessary, this switch enables the use of rituximab, a monoclonal antibody, to target and eliminate the affected cells. It has been demonstrated that UCARTCS1 has significant anti-MM activity, as evidenced by its effects on MM cell lines, primary mPCsandin vivo in a MM mouse model [62]. The activity of UCARTCS1 was found to be independent of the proportion of tumor cells, the SLAMF7 expression level on MM cells, or the frequency of regulatory T-cells in MM bone marrow samples. Furthermore, UCARTCS1 cells exhibited equivalent antitumor activity in samples from both newly diagnosed and heavily pretreated patients, indicating that mechanisms of resistance to prior therapies did not result in diminished susceptibility to UCARTCS1-mediated lysis. The authors evaluated the potential of UCARTCS1 to induce on-target/off-tumor toxicity. According to the data presented, UCARTCS1 exerts its cytotoxic effects on a subset of B cells, NK cells, and T cells that exhibit the highest levels of SLAMF7, while demonstrating minimal impact on cells with low or no SLAMF7 expression. The impact of UCARTCS1 on CD8+ T-cells was more pronounced than on CD4+ T-cells, which is likely related to the lower SLAMF7 expression on CD4+ T-cells. These results are consistent with the published data, which show fratricide killing of CD8+ T-cells during the CAR T-cell manufacturing process when SLAMF7 is not genetically deleted. Indeed, SLAMF7 knockout proved instrumental in preventing CD8+ T-cell fratricide effect during the production process, thereby enhancing the CD4+/CD8+ T-cell ratio in the final CAR T-cell product. The manipulation of the SLAMF7 gene may result in the production of less differentiated cells, as T-cells undergo reduced antigenic stimulation during the manufacturing process. In conclusion, the authors demonstrated that gene editing did not impact the cytotoxic capacity of SLAMF7-targeting CAR T cells. 

Preliminary data from the phase 1 MELANI-01 trial (NCT04142619) presented at the 2021 American Society of Gene and Cell Therapy Annual Meeting [63] indicate that UCARTCS1A demonstrated early antitumor activity in patients with RR MM after failure of CAR T-cell therapy and/or transplant. The expansion and persistence of UCARTCS1A have been observed to correlate with clinically meaningful antimyeloma activity and serum cytokine changes in patients who have undergone extensive pretreatment. The allo-CAR T-cell product consistently remained present in patients, regardless of donor and batch. These preliminary data validated SLAMF7 as a target for allo-CAR T cells. A notable advantage of employing an allo-CAR T-cell over an auto approach is the accessibility of a ready-to-use product. The process of rapid manufacturing has been demonstrated to reduce expenses and yield more than 100 doses from a single batch of donor cells. T cells collected from healthy donors are likely to be more potent because these individuals have not been exposed to high doses of steroids, chemotherapy, or undergone an autologous transplant. However, the Phase I MELANI-01 trial was subject to a clinical hold by the FDA, which was initiated in July 2020 following the occurrence of a fatal cardiac event in a patient, and no results are published. The hold was lifted in November 2020 following the implementation of safety protocol adjustments to the study by Cellectis. Notwithstanding this course of action, the sponsor elected to bring the study to a close, a decision that was made based on its own internal considerations. It is noteworthy that this decision was not influenced by any concerns about safety [64].

Allogeneic CAR-T therapy has several challenges, including issues with managing toxicity. The risk of graft-versus-host disease (GvHD) requires additional genetic modifications, such as TRAC knockout, which can complicate manufacturing and introduce new failure modes [65]. Maintaining cell quality while manufacturing at scale is technically challenging. Immunogenicity concerns may necessitate immunosuppression protocols that could compromise efficacy. Engineering cells to evade immune rejection while preventing GvHD is a delicate process that may compromise CAR-T persistence, a key driver of durable responses.

### 5.3. Dual-Targeting Strategies

Moares et al. developed the CARtein platform, a CAR system that utilizes split inteins to precisely link CAR modules, allowing for the creation of structurally seamless, dual-targeting CARs against BCMA and SLAMF7 [43]. This innovative protein splicing approach enhances the modular design of CARs, which was further refined using advanced protein structure prediction software. The result is a CARtein construct that exhibits robust and precise T-cell activation against cancer cells expressing these antigens. The CARtein platform offers a versatile and potent strategy to overcome limitations in current CAR T-cell therapy, with the potential to improve the safety and effectiveness of treatments for MM. A phase 1/2 trial of a dual-targeted CAR with anti-BCMA and anti-SLAMF7 domains in 16 patients showed an overall response rate of 81% with 38% complete responses, and a 1-year duration of response of 56% [66]. Toxicities included cytokine release syndrome in 38% of patients (grade ≥3 in 6%), no immune effector cell-associated neurotoxicity syndrome, but significant cytopenias in 100% (grade ≥3 in 100%), and infections in 38%. Other groups have developed different CAR constructs co-targeting BCMA and SLAMF7, and one is being clinically tested (NCT0595011) [67,68].

Researchers developed a dual-CAR T cell (DCAR) therapy by targeting CD38 and SLAMF7 on MM cells. In this therapy, CRISPR/Cas9 was used to remove the CD38 gene from T cells to prevent on-target toxicity [69]. The efficacy of the modified DCAR system has been demonstrated in laboratory and animal studies, with the study showing potent anti-MM activity. In comparison to conventional anti-CD38 CAR T cells, the edited DCAR system offers enhanced safety by reducing adverse reactions to normal hematopoietic cells. The engineered T cells are equipped with two CARs to recognize and bind to two distinct antigens, CD38 and SLAMF7. The CD38 gene is deleted from the T cells using CRISPR/Cas9 technology. This deletion renders the engineered T cells incapable of reacting to CD38 on their own surface or on other immune cells (such as NK cells and erythrocytes) and normal tissues. Consequently, this results in an improvement to the safety profile of the therapy.

## 6. Expert Opinion

MM is characterised by phenotypic heterogeneity, suggesting that targeting a single antigen with immunotherapeutic approaches may not be the optimal choice. Presently, anti-BCMA CAR-Ts represent the sole target employed in clinical practice. The expression of BCMA on MM cells is subject to variation, as evidenced by the emergence of BCMA-negative relapses and the presence of MM cells expressing low levels of BCMA prior to anti-BCMA therapy [25,70].

SLAMF7 targets have been identified as a promising alternative antigen due to their absence in both epithelial tissues and hematopoietic stem cells Furthermore, over 95% of bone marrow MM cells express SLAMF7, and this expression persists throughout relapse and treatment with standard therapies. SLAMF7 is expressed on a subset of CD8+ T cells, NK cells, thus its targeting can lead to the depletion of these leukocyte subpopulations and potentially increase the risk of infectious sequelae. The preliminary results of the ongoing trials demonstrate that anti-SLAMF7 CAR-Ts exhibit a significantly enhanced potency against MM cells compared to the anti-SLAMF7 antibody elotuzumab (ELO) [53].

Several relevant unanswered questions regarding SLAMF7 CAR T-cell therapy in MM persist, including the long-term efficacy and durability of the therapy, as well as comparative analyses of its efficacy, safety profiles, and patient quality of life with those of other available therapies. Long-term durability can be assessed by extended follow-up in trials and monitoring CAR-T persistence.

To develop effective treatment strategies, it is essential to understand the mechanisms of resistance and relapse. This includes investigating whether mPCs downregulate SLAMF7 expression or develop alternative escape mechanisms, and how the tumour influences these processes. Furthermore, SLAMF7 expression has been observed on specific immune cells, which has the potential to result in on-target, off-tumour toxicity. Consequently, there is a necessity to analyse strategies that can be employed to enhance the safety profile, such as affinity tuning or controlled CAR expression. To enhance safety, particularly in terms of the risk of infection, the anti-SLAMF7 CAR-Ts currently under investigation incorporate a suicide gene. TALEN gene editing is a technology that has the capacity to suppress endogenous TCR and SLAMF7 expression, thereby mitigating the risk of GVHD and fratricide killing [71]. TALENs require a complex and time-consuming process of protein engineering in order to create custom DNA-binding domains for each target sequence. This makes them costly and challenging to produce on a large scale. The process of protein engineering has been made much easier and faster by CRISPR, which has simplified the process from RNA-coding problems [72]. However, achieving high specificity remains challenging, as does the delivery of CRISPR components. Although CRISPR has transformed gene editing by significantly reducing the barriers to entry for many applications, successfully implementing it in a clinical setting requires overcoming the challenges of off-target effects and efficient delivery [73]. TALENs remain a viable option for specific applications where their precision outweighs their complexity. Ongoing research into CRISPR is making it a more powerful and viable tool for manufacturing therapeutic agents, including those for fratricide.

However, the toxicity profile is different from BCMA-CAR-T, and the on-target/off-tumor effects on immune cells are a key differentiator. The fundamental distinction is that SLAMF7-CAR-T causes immunosuppression through direct depletion of effector immune cells (NK, CD8+ T cells), while BCMA-CAR-T causes immunosuppression primarily through B-cell depletion and hypogammaglobulinemia. 

Furthermore, it is essential to understand the optimal cellular dose and infusion schedule, as well as the methods for streamlining production to ensure wider availability and cost-effectiveness. It is recommended that future studies investigate the immunosuppressive microenvironment in myeloma and its impact on the persistence of CAR T cells. Furthermore, such studies should identify combinations or modifications that can enhance activity within this environment.

A pivotal consideration is the selection of patients; if there are biomarkers (e.g., soluble SLAMF7, genetic markers) that predict responsiveness, it is also important to ascertain which patient populations (e.g., RR vs. newly diagnosed) wilbenefit for the most.

Another open issue is whether SLAMF7 CAR T can be combined with other immunotherapies, such as checkpoint inhibitors or monoclonal antibodies to improve outcomes, and what preclinical or clinical evidence supports combination approaches.

Early-phase SLAMF7-CAR-T trials would likely target a carefully selected patient population to maximize safety assessment while demonstrating proof-of-concept efficacy. Ideal candidates would be patients who have received three to four prior lines of therapy, including proteasome inhibitors, immunomodulatory drugs and anti-CD38 antibodies, and who have progressive disease despite standard treatments, measurable disease by serum/urine M-protein or serum free light chains, and refractory disease to BCMA-targeted therapy. They would also have prior exposure to BCMA-directed therapies (CAR-T, bispecific antibodies, or ADCs), and disease progression during or after BCMA therapy. Confirmed SLAMF7 expression on myeloma cells would be demonstrated by flow cytometry or immunohistochemistry. This population would enable careful assessment of on-target/off-tumour effects (NK and T cell depletion) while addressing the unmet need of BCMA-refractory disease, where SLAMF7 remains a viable target. 

## 7. Conclusions

MM is an active area of clinical research, and, in addition to both mild and aggressive chemotherapy regimens, new immunotherapies are being investigated and integrated into the standard of care. The convergence of robust preclinical data, novel manufacturing methods, and initial clinical safety indications establishes SLAMF7 as a promising alternative or supplementary target to BCMA for myeloma CAR-T therapy. SLAMF7 is uniformly expressed on malignant plasma cells in newly diagnosed myeloma and is still present in relapsed myeloma after intensive therapy, making it an ideal target. SLAMF7 expression on myeloma cells is consistently high and is not affected by cytogenetic abnormalities, genomic mutations, or disease stage. Importantly, SLAMF7 is not expressed in any other normal human tissue outside the haematopoietic system, which reduces concerns about off-tumour toxicity. However, success will depend on rigorous clinical validation, toxicity mitigation, and strategic positioning within the evolving immunotherapy landscape.

## Figures and Tables

**Table 1:** SLAMF7 as a CAR-T Target in multiple myeloma.

PRO		CON		Potential Solutions
High expression in MM cells	Expressed in >95% of MM cells, including in R/R patients. Consistent and stable expression over time	Expression on normal immune cells	It is also expressed on NK cells, CD8+ T cells, B cells, and some dendritic cells. CAR-Ts targeting SLAMF7 may kill endogenous immune cells, leading to immune suppression, T-cell fratricide, or T-cell exhaustion during manufacture.	SLAMF7 knockout in the CAR-T product (most direct solution to fratricide). Careful monitoring of NK cell counts and immune function. Combination approaches with other MM targets to reduce reliance on SLAMF7 alone. Patient selection based on disease burden and immune status
Clinically validated target	Well-studied biologically in MM, reducing unknowns compared to novel antigens. It is the target of elotuzumab, which has a safety profile,	Fratricide and manufacturing complexity:	It is expressed on T cells themselves, which leads to fratricide (CAR-Ts killing each other). This requires gene editing (e.g., CRISPR/Cas9 or shRNA) to knock out SLAMF7 in the CAR-T product, adding complexity, cost, and potential risks.	Virus-Free Manufacturing (Sleeping Beauty System) Gene Editing to Knock Out SLAMF7 Natural Phenotype Acquisition
Lack of expression on HSCs	Unlike CD38 or CD138, SLAMF7 is not expressed on HSCs, thereby preserving hematopoietic reconstitution.	Modest clinical data:	SLAMF7 CAR-Ts have shown some preclinical promise, but clinical trials are limited and mostly in early phases. Some studies paused or terminated due to manufacturing or safety challenges.	Waiting for phase II trials
Possible use in dual-targeting CARs	SLAMF7 can be part of bispecific CAR-T strategies (e.g., SLAMF7 + BCMA), which may reduce escape and enhance durability.	Functional concerns	SLAMF7 may have co-stimulatory roles in T and NK cells. Eliminating SLAMF7+ immune cells could dampen immune surveillance and anti-MM immunity over time.	The success of dual-targeting CARs depends on carefully balancing specificity, activation strength, and T-cell persistence through rational design and iterative optimization based on functional assays.

RR MM (relapsed and refractory Multiple Myeloma); HSCs (hematopoietic stem cells).

**Table 2:** Targeting molecules other than BCMA in Multiple Myeloma.

Target	Characteristics	PRO	CON	Clinical Status/ Considerations
CD138	also known as syndecan-1, is a cell surface protein that plays a significant role in MM	High expression on mature plasma cells and most MM cell—good tumor coverage in many patients. Well-characterized antigen used diagnostically in MM and not expressed on T cells or most hematopoietic stem/progenitor cells—no fratricide and low risk of long-term bone marrow aplasia from target expression on HSCs. Targeting CD138 will deplete malignant plasma cells directly (and normal plasma cells), which is therapeutically beneficial for disease control.	On-target/off-tumor toxicity: also expressed on some normal epithelial cells (e.g., specific mucosal/skin/epithelial compartments). Soluble/shed antigen: Syndecan-1 is shed into the circulation in MM (sCD138). Soluble CD138 can act as a decoy, lowering effective CAR binding, impairing tumor engagement, or causing inappropriate activation/exhaustion/tonic signaling. CD138 expression can be heterogeneous between clones and can be downregulated	Limited clinical experience; potential for poor CAR T persistence/efficacy against marrow niches.
CD19	cell surface glycoprotein traditionally expressed on B cells and their precursors.	Large clinical experience in B-ALL and NHL. Potential to eradicate tumor-initiating clones. Useful in combination/sequential regimens: CD19 CARs can be administered after cytoreduction (e.g., post-ASCT) or combined with BCMA/GPRC5D CARs or bispecific strategies to reduce antigen-escape risk. Predictable, manageable toxicities.	Limited antigen expression on bulk disease: Most myeloma plasma cells lack CD19, so single-agent CD19 CAR T usually has limited direct anti-myeloma activity for the bulk tumor. The benefit is restricted to a minority of patients with measurable disease.	Clinical experience is limited to case reports, small pilot cohorts, and combination/sequential approaches.
CD38	A prominent cell surface glycoprotein that plays a critical role in MM therapy.	High and frequent expression on MM plasma cells—good target coverage in many patients. Well-validated antigen biologically and clinically (therapeutic target of daratumumab/isatuximab)—a known safety/biology profile helps with risk assessment. Not restricted to late B-cell stages only, so can target a broad tumor population, including some CD19-clones. Off-the-shelf approaches feasible (CAR NK, transient mRNA CARs) have been developed to reduce long-term toxicity.	On-target/off-tumor toxicity	Most data are from early-phase studies (preclinical, first-in-human/phase I) with small cohorts. Reports show feasibility and some anti-myeloma activity, but safety concerns and durability remain an open issue
CD56	also known as NCAM (Neural Cell Adhesion Molecule), is a cell surface protein with specific characteristics in MM	Expressed on a large fraction of MM.CD56 is a cell-surface molecule that serves as a biologically accessible antigen for CAR binding.CD56 can be used in patients who relapse after BCMA-directed therapies or in dual-target approaches (BCMA + CD56) to reduce antigen-escape risk.	Widely expressed in the nervous system and some neuroendocrine cells—risk of severe neurotoxicity, peripheral neuropathy, or autonomic dysfunction. NK cells and some T/NKT subsets express CD56; CAR T may deplete NK cells, causing impaired innate immunity and increased infection risk. T cells can upregulate CD56 during activation—CAR T cells may kill each other during expansion, reducing yield and function. Heterogeneous/absent expression on some myeloma clones—antigen escape Not all myeloma cells express CD56 or express it uniformly, which increases the risk of relapse from CD56—negative subclones. Variable antigen density/low target density	Minimal clinical data. Most work was preclinical or at the IND/early-phase planning stage due to serious safety concerns.
NKG2D Ligands	It is a crucial activating receptor in the immune system, primarily found on natural killer (NK) cells and specific T cell subsets	Upregulated in tumor and infected cells, not healthy ones. Broad expression. It can recognize multiple ligands via one receptor with a broad coverage.	It can be upregulated in normal tissues during inflammation with potential off-tumor effects. Soluble MICA/B can act as decoys, suppressing CAR-T function. MM cells can downregulate NKG2D ligands to escape immune detection.	Limited clinical validation; unclear clinical safety profile
MUC1	Mucin1 or MUC1 (also known as CD227, EMA, MCD, MAM6, PEM, PUM, KL-6, CA 27.29/CA 15-3) is a highly glycosylated transmembrane mucin family member protein that is overexpressed in various solid or hematological cancers.	Overexpressed in MM, e specially in advanced or high-risk disease. Tumor-associated isoform (MUC1-C): this isoform is more selectively expressed in cancer cells and can be specifically targeted. Linked to drug resistance and stemness: targeting MUC1 could eliminate more aggressive or resistant clones.	Expression on normal epithelial cells. Glycosylation heterogeneity	Limited clinical data. Mostly preclinical or early-phase trials.
GPRC5D	Class C orphan G protein-coupled receptor is predominantly expressed in MM cells and hard keratinized tissues, with low expression in normal human tissues.	Uniformly and highly expressed in MM, even in patients who relapse after BCMA CAR-T. Low expression in normal tissues. It shows vigorous activity, including post-BCMA relapse.	On-target off-tumor toxicity: Nail, skin, taste-related side effects (e.g., dysgeusia, rash, nail changes), although generally manageable. Bispecifics competition.	The most clinically mature target. Demonstrates strong efficacy, even in patients with BCMA-refractory disease.
CD229	Also known as Ly9, it is a cell surface receptor belonging to the SLAM family.	Broad expression: Many studies (preclinical and translational) report high and relatively uniform CD229 expression on malignant plasma cells, including some clones with low BCMA. This can increase tumor coverage and reduce the risk of antigen escape when used alone or in combination. Potential to target progenitor/subclone population	On-target/off-tumor toxicity: CD229 is expressed on normal lymphoid cells (B cells, subsets of T cells, and possibly NK/other hematopoietic cells). Fratricide and manufacturing difficulty. Immune dysfunction and dysregulation. Prolonged immune suppression. Antigen heterogeneity/escape. Soluble forms or modulation of CD229 are not well characterized; the potential impact on CAR function is uncertain.	Most data are preclinical or early translational; clinical toxicity spectrum, durability, and real-world feasibility are not well established.
APRIL	A Proliferation-Inducing Ligand, member of the TNF family	High specificity for myeloma cells: APRIL binds to BCMA, TACI, and BAFF-R, which are frequently overexpressed on MM plasma cells, providing potentially broad coverage. Dual receptor targeting Potential: By using APRIL-based ligands or diminished affinity CARs, the approach can target multiple receptors	APRIL interacts with receptors on normal immune cells, including B cells and other lymphocytes, thereby raising the risk of on-target/off-tumor toxicity (e.g., B cell aplasia, immune dysregulation). Variability in receptor expression levels may result in antigen escape or reduced efficacy.	APRIL-based CAR T cells have shown effective cytotoxicity against myeloma cells in preclinical models.
κ Light Chain	Components of immunoglobulins. MM cells express either κ or λ light chains, but not both.	MM express only one type of light chain (κ or λ), targeting κ allows selective killing of malignant cells while sparing normal B cells expressing the other type. It can be customized per patient based on the expressed light chain. Reduces the risk of total B-cell aplasia	Only ~60% of myeloma cases are κ-restricted. Therapy is not suitable for λ+ myeloma. Antigen escape can occur via downregulation or mutation. Some normal B cells expressing κ will still be targeted, leading to partial B-cell aplasia.	Useful for κ+ MM patients with minimal B-cell aplasia
CCR10	Chemokine receptor involved in plasma cell homing to bone marrow and mucosal tissues, and overexpressed in some MM subtypes.	Targeting CCR10 may disrupt tumor localization or retention in the bone marrow niche, potentially leading to tumor-selective targeting.	Not universally or uniformly expressed in all MM patients. Some expression in mucosal tissues (e.g., skin, gut), raising concerns about off-tumor effects.	Limited clinical validation
CD44v6	It is a cell surface glycoprotein involved in cell adhesion and migration. The v6 isoform is selectively expressed in several cancers, including MM.	More specific to malignant plasma cells than CD44 (ubiquitous). CD44v6 is associated with poor prognosis and disease aggressiveness.	Expression in normal keratinocytes (related to cutaneous side effects). Not all MM cells express CD44v6, which may lead to immune escape. Variable expression due to alternative splicing may affect durability and efficacy.	Encouraging results in MM and AML. Novel mechanisms, such as targeting the microenvironment or aggressive subtypes
FcRH5	A B-lineage-specific protein expressed on normal and malignant plasma cells, including MM	High expression in MM. Limited to the B-cell lineage, reducing off-target effects.	Low expression on some normal B cells. Myeloma cells may reduce FcRH5 expression under selective conditions.	Under clinical evaluation. Relatively safe and promising target
TACI	Transmembrane Activator and CAML Interactor, also known as TNFRSF13B, is a receptor expressed on specific B cells and plasma cells.	Frequently overexpressed on MM plasma cells, providing a relevant target for cell therapy. It may help target BCMA-negative or low-expressing clones. TACI is found on some memory B cells, which may support immune reconstitution after therapy.	It is present on normal B cells and some immune regulatory cells, raising concerns about on-target/off-tumor toxicity, including B cell aplasia and immune deficiency. Safety concerns: Off-tumor toxicity could result in Potential for antigen escape: Tumor cells may downregulate TACI, leading to relapse.	TACI-targeted CAR T therapies are primarily in preclinical or early-stage trials, resulting in limited safety, efficacy, and durability data.
Integrin β7	Integrin β7 is a cell adhesion molecule involved in the regulation of immune cell migration and adhesion. In the context of MM, it plays a role in the homing and retention of plasma cells within the bone marrow microenvironment.	Selective expression in MM. Plays a role in MM homing and interaction with the bone marrow, and targeting may disrupt microenvironmental support. Expression is limited primarily to lymphoid tissue, with potentially less systemic toxicity.	Integrin β7 is involved in lymphocyte homing, allowing for potential off-tumor effects. The long-term expression and resistance dynamics are not well understood.	Clinical data in preclinical or early-phase
